# Robust clothing-independent gait recognition using hybrid part-based gait features

**DOI:** 10.7717/peerj-cs.996

**Published:** 2022-05-31

**Authors:** Zhipeng Gao, Junyi Wu, Tingting Wu, Renyu Huang, Anguo Zhang, Jianqiang Zhao

**Affiliations:** 1 Xiamen Meiya Pico Information Co., Ltd., Xiamen, Fujian, China; 2 College of Mathematics and Data Science, Minjiang University, Fuzhou, China; 3 College of Physics and Information Engineering, Fuzhou University, Fuzhou, China

**Keywords:** Gait recognition, Part-based, Spatio-temporal feature learning, Clothing-independent

## Abstract

Recently, gait has been gathering extensive interest for the non-fungible position in applications. Although various methods have been proposed for gait recognition, most of them can only attain an excellent recognition performance when the probe and gallery gaits are in a similar condition. Once external factors (*e.g.,* clothing variations) influence people’s gaits and changes happen in human appearances, a significant performance degradation occurs. Hence, in our article, a robust hybrid part-based spatio-temporal feature learning method is proposed for gait recognition to handle this cloth-changing problem. First, human bodies are segmented into the affected and non/less unaffected parts based on the anatomical studies. Then, a well-designed network is proposed in our method to formulate our required hybrid features from the non/less unaffected body parts. This network contains three sub-networks, aiming to generate features independently. Each sub-network emphasizes individual aspects of gait, hence an effective hybrid gait feature can be created through their concatenation. In addition, temporal information can be used as complement to enhance the recognition performance, a sub-network is specifically proposed to establish the temporal relationship between consecutive short-range frames. Also, since local features are more discriminative than global features in gait recognition, in this network a sub-network is specifically proposed to generate features of local refined differences. The effectiveness of our proposed method has been evaluated by experiments on the CASIA Gait Dataset B and OU-ISIR Treadmill Gait Dataset B. Related experiments illustrate that compared with other gait recognition methods, our proposed method can achieve a prominent result when handling this cloth-changing gait recognition problem.

## Introduction

For decades, there has been a growing demand for robust surveillance applications. Given that each person reveals his/her biometric features, *e.g.*, face, iris, fingerprint, gait, *etc*, in a sufficiently characteristic and fairly individual way, lately recognition using biometrics has been widely utilized in most surveillance systems. However, in the real world the captured surveillance images can be influenced by low resolution, poor illumination, *etc*. Monitoring targets also can cover their most regular biometrics by using masks, glasses, and gloves. Thus, a biometric feature, which shows more robust to these factors, is more popular for real-world surveillance systems, *e.g.*, gait.

Compared with other biometric features, recognition using gait offers a better option for most surveillance systems. First, it is difficult to disguise other people’s gaits, because walking is essential for human mobility. Second, gait can be measured from a distance without physical contact or proximal sensing. Third, gait works well in an unconstrained scenario. It can be recognized from a single still image or a sequence of continuous frames. All these strengths makes gait specifically attractive for human authentication. In Denmark and the UK, gait analysis plays an important part for evidence collection when convicting criminals (Bouchrika et al., 2011; Iwama et al., 2013; Wu et al., 2017; Zhang et al., 2021).

The potential of gait recognition is enormous, but currently gait recognition is more at the evaluation stage rather than the application stage; thus, gait analysis is still in the infancy (Makihara, Nixon & Yagi, 2020). Gait recognition is one of the newest biometric authentication methods, since its development truly begins as the processing speeds and the computer memories became sufficient to settle gait sequences with a considerable performance (Makihara, Nixon & Yagi, 2020).

Meanwhile, although lately many gait recognition methods have been proposed, most of these methods can only attain an outstanding recognition performance when the probe gait and the gallery gait are in a similar environment (Makihara, Nixon & Yagi, 2020; Liu, Liu & Zhang, 2022). It becomes more challenging when people’s gaits are impacted by other factors and the probe/gallery gaits are no longer similar. Examples of factors that will cause a negative influence on gait recognition are: clothing variations (Hossain et al., 2010; Deng & Wang, 2018; Anusha & Jaidhar, 2019; Yao et al., 2021a), carrying bags (Singh & Biswas, 2009; Zhang, Wu & Xu, 2019b; Yoo & Park, 2021), walking/running modes (Kusakunniran et al., 2012b; Yao et al., 2022; Makihara et al., 2018), *etc*. There also remain some other influencing factors which are relevant with the external environment. Examples of these factors are: view angle changes (Kusakunniran et al., 2012c; Iwashita, Ogawara & Kurazume, 2014; Yao et al., 2021), *etc*. Among these factors, clothing variations can be seen as the most challenging factor for gait recognition (Hossain et al., 2010; Yao et al., 2021c). Thus, in this article, an efficient gait recognition method is proposed to handle this cloth-changing problem.

For gait, different body parts are differently influenced by clothing variations. There remain many body parts which are significantly influenced by clothing variations, and there also remain lots of parts which still can retain relatively unchanged regardless of the cloth-changing influence (Hossain et al., 2010; Yao et al., 2021c; Zhang & Wang, 2022). Thus, in our method, we mainly focus on extracting robust gait features from the body parts which are non/less vulnerable to clothing variations. Meanwhile, given that comparison experiments in Wu et al. (2017) have indicated that in gait recognition local detailed features prove more discriminative than global semantic features; thus, in our method we give more attention to the local refined differences within human gaits, and a sub-network is specifically designed to extract more discriminative local spatial features. Also, given that temporal features can be deemed as an effective feature complement to enhance the recognition performance (Fan et al., 2020; Yao et al., 2021b), one sub-network is also specifically used in our method to model the micro-motion temporal relationship among continuous short-range frames. Moreover, related experiments have proved that our proposed hybrid feature learning method can always obtain a prominent performance when approaching this challenging cloth-changing gait recognition problem.

Contributions of this article are summarized as follows,

• This article generates a hybrid part-based spatio-temporal feature for gait recognition to approach the cloth-changing problem. This hybrid spatio-temporal feature is made up of three different parts, each generated by one specific sub-network. Each sub-network emphasizes an individual aspect of gait, thus a robust hybrid gait feature has been generated through their concatenation.
• This proposed method has presented an excellent performance for cloth-changing gait recognition on CASIA Gait Dataset B and OU-ISIR Treadmill Gait Dataset B.

## Related Work

In this section, a brief survey is given for gait recognition.

In decades, a number of different methods have been raised for gait recognition. Roughly, these proposed methods can be classified into two categories, *i.e.*, template-based methods or sequence-based methods (Chao et al., 2019).

### Template-based gait recognition

For template-based methods, a pre-process of integrating gait templates from images/videos is first needed. One of the most commonly accepted templates in this category is Gait Energy Image (GEI) (Han & Bhanu, 2006), integrated by averaging aligned silhouettes within a whole gait cycle. Another similar template is Motion Silhouette Image (MSI) (Lam & Lee, 2006), where each pixel is denoted as a descriptor of its motions in the temporal domain across all the silhouettes which are part of a whole gait cycle. Distinct from GEI and MSI, Skeleton Gait Energy Image (SGEI) is denoted as an average product of human skeleton models over a whole gait cycle (Yao et al., 2018; Yao et al., 2021). Once gait templates are attained, various machine learning methods and deep learning networks can be chosen to extract the representations of gait and enhance their characterization capabilities. Finally, the similarities between gait representations can be matched using Euclidean distance or some other metric learning methods (Wu et al., 2017; Yao et al., 2021; Takemura et al., 2019).

Basically, template-based methods divide this pipeline into two parts, *i.e.*, template generation and matching (Chao et al., 2019). The aim of template generation is to transform gait information across frames into a single gait template (Chao et al., 2019). In this way, both spatial and temporal information have been efficiently embedded for each template. Moreover, the recognition performance is also significantly influenced by the transformed templates. Taking GEI for example: because silhouettes are sensitive to clothing changes, GEI cannot always present a satisfying performance if the probe/gallery gaits are in two varying dressing patterns. Also, given that viewing changes can prominently change the accessible visual features to be used, the recognition accuracy of GEI can go through a significant degradation if the viewing gaps get larger. In order to decline these negative influences, a wide range of machine learning methods have been raised for matching templates (Kusakunniran et al., 2012a; Matin, Paul & Sayeed, 2017; Kusakunniran et al., 2009). For example, in Kusakunniran et al. (2009), an adaptive weighting method was used to distinguish significance of bits for rescaled GEI. In Kusakunniran et al. (2012a), a View Transformation Model (VTM) was proposed to learn the relationship between different views, and a view-invariant gait representation can be learned by projecting GEI into a latent subspace. In Matin, Paul & Sayeed (2017), a method was proposed to detect co-factor affected segments of GEI. GEI is first divided into different parts based on the area of co-factor appearance. Then, co-factored cues are detected and reduced according to the predefined thresholds. Finally, a co-factored GEI is dynamically reconstructed through combination.

Recently, deep learning has been flourishing in computer vision community, and a large number of deep learning-based networks also have been constructed for template-based gait recognition. Shiraga et al. (2016) proposed GEINet with GEI as its input. Zhang et al. (2016) fine-tuned a Siamese neural network for feature generation and used KNN for feature matching. In Li et al. (2020a), an encoder was utilized to disentangle GEI into identity and covariate features. In the decode stage, the original GEI and the canonical GEI without any covariates were both rebuilt. In Zhang, Wu & Xu (2019b), a view transformation generative adversarial network (VT-GAN) was adopted for gait features to achieve transformation across any two views using a single generic model. Further, in Zhang, Wu & Xu (2019a) an identity-preserved variation normalizing generative adversarial network (VN-GAN) was also proposed to formulate identity-related features. For the aforementioned deep learning-based methods, a main disadvantage of using gait templates as input is that they may loss the individual information of each frame, since generally they are generated by stacking and averaging frames together. Also, given that only one or two gait templates can be formed from one sequence, it may lead to the problem of insufficient input training data.

### Sequence-based gait recognition

Different from template-based methods formulating gait templates first, sequence-based methods directly treat a sequence of gait frames as input. Based on the manners of formulating temporal features, these methods can be divided into different categories, *i.e.*, 3D CNN-based and LSTM-based (Chao et al., 2019). A main advantage of these methods is that they can capture individual information for each frame. Also, more temporal information can be formulated since specialized structures are utilized (Chao et al., 2019). In Wolf, Babaee & Rigoll (2016), a 3D-CNN network is proposed to capture features in multiple views. In Lin, Zhang & Bao (2020), a multiple-temporal-scale framework is proposed to model temporal information in multiple scales. In Feng, Li & Luo (2016), heat maps were first explored as features of each frame, then a LSTM network was used to assemble the features of each frame into a feature of the whole sequence. In Battistone & Petrosino (2019), features were first modeled for each skeleton key-point and then attached to the graph/skeleton edges. A LSTM-based network was adopted to jointly exploit structured data and temporal information by learning long short-term dependencies from graph structures. In Zhang et al. (2020), features of human body parts were extracted and linked as input, and LSTM models were handled as temporal attention models to calculate the attention score of each frame. In Zhang et al. (2022), pose, appearance, and canonical features were formulated from input frames first using disentanglement learning. Followed, a LSTM network is adopted to integrate the pose features into a dynamic feature, and the canonical features are averaged as a static feature. All these methods have presented a prominent result for gait recognition in various conditions. However, for these methods, a huge disadvantage is their high computation cost (Chao et al., 2019), which may limit their usage in practical applications.

Recently, some 2D-CNN networks also have been raised to approach this gait recognition problem in the sequence-based way. Different from the aforementioned methods learning the temporal relation between continuous frames, these networks assume that the appearance of each silhouette has involved its positional information, in which way the order information of a sequence is not necessary for gait recognition (Chao et al., 2019). Examples of these networks are Wu, Huang & Wang (2015); Chao et al. (2019); Yao et al. (2021b). In Wu, Huang & Wang (2015), its main network first approached each input frame independently *via* a number of 2D convolution units with shared weights. A global spatio-temporal pooling module was adopted at the top to combine the independent information of each input frame into a feature of the whole sequence. In Chao et al. (2019), a global pipeline was used to collect combined information from different levels. Chao et al. (2019) presented a state-of-the-art performance for gait recognition in different datasets. Moreover, Fan et al. (2020) improved the network proposed in Chao et al. (2019) by using a special module to model the micro-motion patterns within input frames. The performance improvement indicates that although the appearance of each silhouette do contain the positional information, additional temporal modeling can use as a complement to enhance its recognition performance.

## Methods

### Overview

For image/video-based gait recognition, its core lies in extracting robust gait-related features from walking sequences (Zhang et al., 2019). In our article, encouraged by Wu et al. (2017), Feichtenhofer et al. (2019), Chao et al. (2019), Sun et al. (2019) and Fan et al. (2020), a robust hybrid gait-related feature is formulated for cloth-changing gait recognition from each input walking sequence.

**Figure 1:**
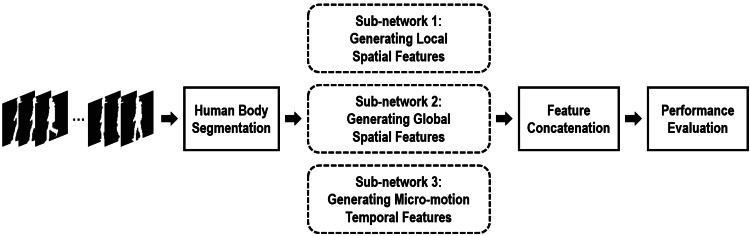
Framework of the proposed method.

Figure 1 reveals the framework of our proposed network. For each input sequence, the silhouette human bodies of each frame are first segmented into the affected parts and the non/less affected parts based on the anatomical studies of gait (Dempster & Gaughran, 1967). Then, focused on the non/less affected human body parts, three different sub-networks are specifically proposed to capture efficient gait-related features. Finally, through assembling these three gait-related features, a feasible hybrid spatio-temporal gait feature has been formulated in our method. Experiments certify that this proposed hybrid gait feature will achieve an excellent result for gait recognition when approaching the cloth-changing problem.

More specifically, assuming a silhouette sequence consisting of *n* successive frames can be represented as *χ* = {*x*_*i*_|*i* = 1, 2, 3, …, *n*}, and its segmented non/less affected human body parts can be represented as $\tilde {\chi }$, thus our proposed hybrid part-based spatio-temporal feature can be learned as,

(1) \begin{eqnarray*}f=H(G({F}_{sl}(\tilde {\chi }))){}H(G({F}_{sg}(\tilde {\chi }))){}H(G({F}_{t}(\tilde {\chi })))\end{eqnarray*}

where *F*_*sl*_ and *F*_*sg*_ denote creating local and global spatial features, respectively, from each non/less affected body part using a variety of 2D convolution units with shared weights. *F*_*t*_ denote grasping the micro-motion gait features from the non/less affected body parts between continuous short-range frames. *G* represents assembling the spatial/motion features of each silhouette into a spatial/motion feature of the entire sequence, and *H* represents the transforming these assembled spatial/motion features into a more discriminative subspace to enhance their discrimination capabilities. ⨁ denotes the feature concatenation operation.

### Segmenting human bodies

In gait recognition, part-based strategies are widely utilized when approaching the cloth-changing problem. For example, in Hossain et al. (2010), the heavier weighting was assigned to the body parts which enable to maintain unchanged by clothing variations, while the lighter weighting is assigned to the other body parts which can be significantly changed. The main reason that part-based strategies work well in cloth-changing gait recognition is that clothing variations generally can cause different influence on different human body parts (Hossain et al., 2010). Hence, for these part-based gait recognition methods, it plays a significant role in accurately segmenting each human body into the affected parts and the unaffected parts.

For our method, each human body is first divided into the affected and non/less affected parts according to the anatomical studies of gait (Dempster & Gaughran, 1967). Assuming a human body of *H* height, we can segment his/her entire body into a series of different parts at some key positions, *e.g.*, neck (0.87*H*), waist (0.535*H*), pelvis (0.48*H*), and knees (0.285*H*) (Dempster & Gaughran, 1967). Moreover, because clothes designing and tailoring are not always totally consistent with the anatomical studies, in our method the segmentation restriction has been moderately relaxed while we segment human bodies. Thus, the final segmented parts are actually a little broader than they ought to be. Furthermore, given that for each person the upper body can be more easily to be influenced by clothing variations, thus in the proposed method we mainly focus on two segmented parts, *i.e.*, the head part and the crus part.

**Figure 2:**
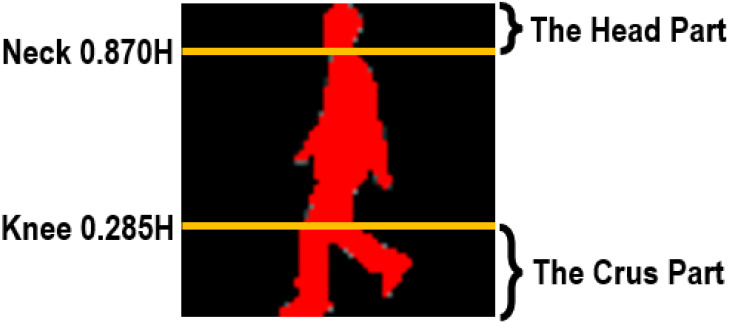
Segmentation of human bodies.

Figure 2 shows a sample of our segmented head and crus parts.

### Generating spatial features

As Fig. 1 reveals, in this article two sub-networks are specially proposed to capture local and global spatial features for cloth-changing gait recognition.

#### Motivations

In Wu et al. (2017), a comprehensive analysis was made on cross-view gait-based human identification using deep CNNs. Three different networks are proposed and compared with a pair input of two GEIs. Among these three networks, the only shared module was two successive units made of one convolution, normalization, and pooling layers, and the major disparity is when and where their extracted gait-related features are compared. In LB-Net, local features were compared at the first layer. In MT-Net, mid-level features were compared at the top layer. In GT-Net, global features were compared at the top after a fully-connected layer. Experiment results illustrate that there are no significant gaps between the performances of LB-Net and MT-Net, while they both outperform GT-Net with a distinct margin (Wu et al., 2017). To some extent, gait recognition can be seen as a fine-grained task, and it highly relies on the existing local subtle differences. Thus, a good gait recognition network can always take full advantage of the refined information within local areas, *e.g.*, LB-Net and MT-Net in Wu et al. (2017). Meanwhile, considering that in a CNN-based network pixels in the feature maps of shallow layers are more related with local fine-grained information while pixels in the feature maps of deeper layers are more connected with global coarse-grained information (Chao et al., 2019), in our method we focus more attention on the features generated from shallow layers.

#### Generating local spatial features

**Figure 3:**
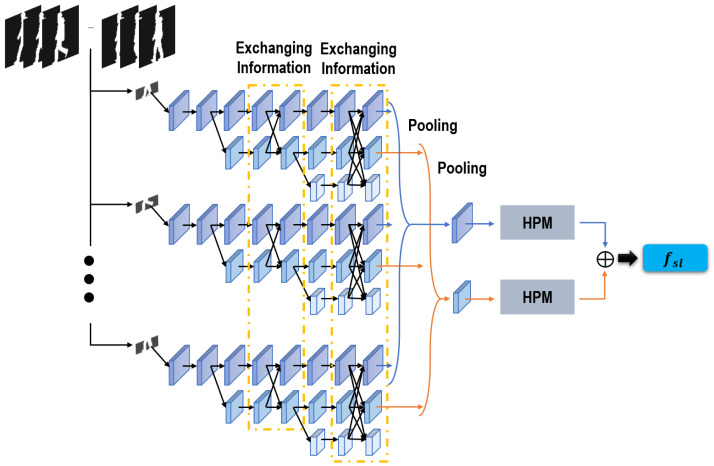
Sub-network used to extract local spatial features.

Figure 3 illustrates the architecture of our sub-network proposed for generating local spatial features. Given that features of the crus part are more efficient than features of the head part (Yao et al., 2021c), in our method we mainly focus on seeking robust local spatial features from our segmented crus parts.

Stimulated by Chao et al. (2019) and Sun et al. (2019), distinct from most gait recognition networks stacking convolutions in turn and capturing features from deeper layers, our network convolutions are placed in parallel, and only features from shallow layers will be learned for the following recognition task. Specifically, as revealed in Fig. 3, starting with a convolution unit as our first convolution stage, we gradually create more convolution stages by adding another convolution unit. After each convolution stage, a sub-network is formed, and all these sub-networks are arranged in parallel. Thus, finally, three convolution stages are created and three parallel sub-networks are formed. The feature scales are gradually reduced as more convolution stages are created, but they will maintain unchanged throughout each following sub-network. Moreover, given that this proposed network enables to learn and preserve multi-scale features for gait recognition, a scheme of information exchanging is also established in this network to enhance the feature robustness. As Fig. 3 presents, information is exchanged over any two sub-networks through the functions of upsampling and downsampling. Through this information exchanging scheme, a more robust feature can be integrated by this proposed network. On the one hand, a more robust global feature can be formed in this network by adding more convolution stages, which can lead the shallow stages to offer a more robust feature representation for the local refined differences. On the other hand, the spatial-aware cues of local areas are well preserved throughout each sub-network, which can lead the deeper stages to formulate a more efficient global semantic feature. In this way, a tendency of mutual utilization and promotion has been integrated in our network, which also improves the robustness of our extracted gait-related features. Besides, given that features of shallow layers are more related with local subtle information, in this network only features of the first two sub-networks are involved in the next recognition task. A pooling operation is used to map these two features of every frame into two features of the entire sequence. HPM (Chao et al., 2019) is also used to project these two sequence features into a more discriminative feature space. Finally, our utilized local spatial features are integrated through the concatenation of these two sequence features.

#### Generating global spatial features

Although local features prove more discriminative than global features for gait recognition (Wu et al., 2017), it is not a practical option to totally ignore global features when identifying different gaits. Local region based features cannot model the relations across neighboring regions, thereby influencing the robustness (Lin et al., 2020). Thus, in our method, another sub-network is designed to make up for the global features ignored in ‘Generating local spatial features’.

Consisting of two branches aiming to tackle our segmented head and crus parts independently, the architecture of the sub-network is much similar to that proposed in Yao et al. (2021c). Each branch includes three convolution stages, and each convolution stage consists of two sequential convolution layers and one pooling layer. After all convolution stages, a pooling operation is used to integrate the features of each frame into a feature of the full sequence. HPM (Chao et al., 2019) is also followed to project these sequence features into a more discriminative feature space. Finally, our required global spatial features are learned by hybridizing the features of the head and crus parts together.

Although global features are also involved when extracting local spatial features, it is rational for us to adopt another sub-network to capture global spatial features. As shown in Fig. 3, a scheme of information exchange is adopted when extracting local spatial features. With this direct connection, its involved global features are highly correlative with its local features of shallow layers. Learning global spatial features using another sub-network can not only preserve the independence, but also achieve the diversity of different features.

### Generating temporal features

As Fig. 1 shows, in our method another different sub-network is specially used to explore robust temporal features for cloth-changing gait recognition.

#### Motivations

The gaits of each individual can be deemed as postures from a set, in a sequence showing an implicit structured probabilistic nature (Sundaresan, Roy-Chowdhury & Chellappa, 2003). Thus, it is reasonable for gait recognition to focus some attention on the temporal correlation across consecutive frames, *e.g.*, HMM in Sundaresan, Roy-Chowdhury & Chellappa (2003), LSTM in Zhang et al. (2019) and GRU in Sepas-Moghaddam et al. (2021). In our method, it is apparent that no temporal features have been explicitly modeled when formulating spatial features, thereby leading to the information loss in time series (Wu et al., 2017; Wang et al., 2012). Thus, in our method another sub-network is proposed to remedy the lost temporal information.

Generally, most gait recognition methods represent the global understandings of gait sequences through modeling the long-range dependencies (Fan et al., 2020). However, for a successive gait sequence, frames with similar appearance are more likely to arise at fixed time intervals, which illustrates that the long-range dependencies, *e.g.*, in most cases longer than a whole gait cycle, may be redundant and inefficient for gait recognition (Fan et al., 2020). Thus, compared with modeling the generally used long-range dependencies, it is more rational and efficient for gait recognition to attach more attention to the short-range dependencies across successive short-range frames.

#### Generating micro-motion temporal features

**Figure 4:**
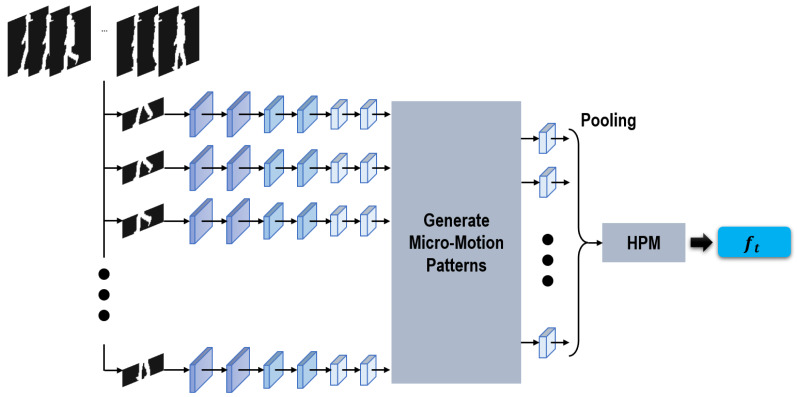
Sub-network used to extract temporal features.

Figure 4 illustrates the architecture of our sub-network proposed for grasping micro-motion temporal features among consecutive short-range frames. Considering that the segmented crus parts enable to generate more temporal cues than the head parts (Yao et al., 2021c), in this part we only focus attention on extractiing micro-motion temporal features from the aforementioned crus parts.

Taking a clip of continuous silhouettes as input, this proposed sub-network first approached each input silhouette independently. After that, inspired by Wu et al. (2017) and Fan et al. (2020), a module is specifically proposed in our sub-network to generate the micro-motion temporal features across continuous short-range silhouettes. Motivated by GEI formed by averaging silhouettes within an entire gait cycle (Han & Bhanu, 2006), in our network the micro-motion temporal features are generated by taking max-pooling operations within successive short-range silhouettes. The max-pooling operations function as sliding-window models, formulating the micro-motion temporal features across short-range silhouettes using a shared max-pooling operation. Furthermore, in order to combine multi-scale temporal information, in our method two different window sizes, *i.e.*, 3 and 5, are utilized. Finally, a pooling operation and HPM are also used as we generate the local/global spatial features in ‘Generating spatial features’.

It is worth noticing that there remain significant differences between Fan et al. (2020) and our proposed method. For Fan et al. (2020) the micro-motion patterns were formulated at the top following HPM, while in this method they are directly formulated from the feature maps before HPM. In this way, more refined local motion information can be retained in our formulated micro-motion temporal features.

### Other details

As Fig. 1 indicates, in this method our hybrid gait-related features used for recognition are formulated by concatenating features of each sub-network together. Batch All (*BA*_+_) triplet loss is also used in this method to train our proposed network (Hermans, Beyer & Leibe, 2017).

## Experiments

In this section, we validated the robustness of our proposed method on two of the most widely-used datasets, the CASIA Gait Dataset B (Zheng et al., 2011) and OU-ISIR Treadmill Gait Dataset B (Makihara et al., 2012). Our training and testing details are first shown in ‘Training and testing details’. After that, more details about these two comparisons are shown in ‘Comparison experiments on CASIA gait dataset B’ and ‘Comparison experiments on OU-ISIR treadmill gait dataset B’. Finally, ablation experiments are given in ‘Ablation experiments on CASIA gait dataset B’. These comparison experiments illustrate that compared with other gait recogition methods, our proposed method can achieve a more robust performance when handling the cloth-changing problem.

### Training and testing details

For our experiments, the input is aligned silhouette sequences in size of 64 × 64. In the training stage, a clip of 30 silhouettes is first randomly intercepted from each sequence. After that, a batch with size of 8 × 8 is sampled from each training dataset, which indicates that each batch contains eight persons and each person can have eight clips in each batch. The parameters of each sub-network are revealed in Table 1. Adam (Kingma & Ba, 2015) serves as our optimizer, with its learning rate set to be 1*e* − 4. The margin in *BA*_+_ triplet loss (Hermans, Beyer & Leibe, 2017) is set to be 0.2. In the testing stage, in order to suppress the uncertainty of random sampling, each batch size is set as 1, and the full silhouette sequences are adopted as our testing input.

**Table 1:** Sub-network parameters.

Sub-network	Convolution channels	HPM scales
Sub-network 1	$ \left\{ 64,128,256 \right\} $	{5, 4}
Sub-network 2	$ \left\{ 32,64,128 \right\} $	{2, 4}
Sub-network 3	$ \left\{ 32,64,128 \right\} $	4

### Comparison experiments on CASIA gait dataset B

CASIA Gait Dataset B (Zheng et al., 2011), one of the most widely-used gait datasets, captures gait data from 124 persons under 11 different viewing angles (0°, 18°, 36°, …, 180°). For each person under each viewing angle, 10 videos are provided, 6 videos in normal styles (NM#1-6), 2 videos with a long coat (CL#1-2), and 2 videos with a bag (BG#1-2). Gait silhouettes are also directly offered by this dataset. In our experiments, all silhouettes are aligned using the method in Chao et al. (2019).

Our comparison experiments on this dataset include three different parts.

In the first part, only clothing changes have been taken into account. Under each view, the training set is assembled by the first three NM videos (NM#1-3) and the first CL video (CL#1) of all persons. Also, under each view, two testing sets are evaluated, the first comprised of the left three NM videos (NM#4-6), and the other comprised of the left CL videos (CL#2). Table 2 exhibits the experiment results for our method and three other gait recognition methods under the common viewing angles. From this table, we can find that this proposed method exceeds other gait recognition methods with an obvious margin. In all cases, our proposed method has achieved the accuracy of 100%. This experiment certifies that compared with other gait recognition methods, under a fixed common viewing angle our proposed method is more efficient and robust to handle the cloth-changing gait recognition problem.

**Table 2:** Comparison on CASIA-B under the same viewing angle by accuracies (%).

Probe set	**Ours**	Yao et al. (2021c)	Anusha & Jaidhar (2019)	Deng & Wang (2018)
36° (NM)	**100.0**	**100.0**	90.5	89.5
36° (CL)	**100.0**	**100.0**	90.9	91.1
54° (NM)	**100.0**	**100.0**	91.1	88.2
54° (CL)	**100.0**	**100.0**	93.2	91.9
72° (NM)	**100.0**	**100.0**	94.7	88.7
72° (CL)	**100.0**	**100.0**	96.5	89.5
90° (NM)	**100.0**	**100.0**	93.5	87.1
90° (CL)	**100.0**	99.2	95.1	88.7
108° (NM)	**100.0**	**100.0**	92.7	–
108° (CL)	**100.0**	99.2	94.1	–
126° (NM)	**100.0**	**100.0**	91.1	–
126° (CL)	**100.0**	**100.0**	91.5	–
144° (NM)	**100.0**	**99.7**	92.2	–
144° (CL)	**100.0**	**100.0**	93.5	–

**Notes.**

The first and second highest scores are represented by bold and underline, respectively.

In the second part, both variations of views and clothes have been taken into our consideration, and an unconstrained environment has been simulated for performance evaluation. As Table 3 illustrates, in this part six probe/gallery view pairs are simulated within the common viewing angles (Chen et al., 2018). For each probe/gallery view pair (*θ*_*p*_, *θ*_*g*_), the training set is formed by videos of the first 34 persons under the 2 viewing angles of *θ*_*p*_ and *θ*_*g*_. For testing, the two CL videos of the left 90 persons under the viewing angle of *θ*_*p*_ are regarded as the probe, and the six NM videos of the left 90 persons under the viewing angle of *θ*_*g*_ are regarded as the gallery. Table 3 reveals the comparison results of our proposed method and four other gait recognition methods. It is evident that our proposed method has obtained the best recognition performance in this unconstrained environment. Its mean accuracy peaks at 96.6%, outperforming Yao et al. (2021c) by 1.0%. This comparison experiment illustrates that although our method is not designed for gait recognition to approach the view-changing problem, it still indicates a strong robustness against viewing changes. Therefore, it can be concluded that compared with other gait recognition methods, our proposed method has a promising application in real-world surveillance systems.

**Table 3:** Comparison on CASIA-B under different walking conditions by accuracies (%).

(Probe, Gallery)	**Ours**	Yao et al. (2021c)	Zhang et al. (2019)	Chen et al. (2018)	Wu et al. (2017)
(36°, 54°)	**97.2**	93.7	87.0	59.8	49.7
(54°, 72°)	**97.2**	94.1	90.0	72.5	62.0
(72°, 90°)	97.2	**98.8**	94.2	88.5	78.3
(90°, 108°)	97.8	**98.7**	86.5	85.7	75.6
(108°, 126°)	**95.0**	94.9	89.8	68.8	58.1
(126°, 144°)	**95.0**	93.5	91.2	62.5	51.4
Mean	**96.6**	95.6	89.8	73.0	62.5

**Notes.**

The first and second highest scores are represented by bold and underline, respectively.

In the final part, our proposed method is compared with the state-of-the-art deep learning-based gait recognition methods in the LT setting (Chao et al., 2019). The training set consists of videos of the first 74 persons, and the testing set is made up of videos of the left 50 persons. The two CL videos are handled as the probe, and the first four NM videos are tackled as the gallery. Table 4 shows the comparison of our proposed method and some state-of-the-art gait recognition methods. Results offered in Table 4 are averaged on the gallery views, and all identical views have been excluded from each averaging process. It can be seen that this proposed method has presented a remarkable performance in the LT setting, attaining the second best recognition result. Moreover, except the front view(0°) and the back view (180°), our proposed method has always achieved the top three recognition accuracies under each view. The main reason why our proposed method is a little inferior than Fan et al. (2020) lies in that for Fan et al. (2020) a channel-wise attention function is proposed to re-weight the feature vectors among micro-motion patterns while in our method all feature vectors are equally processed. A more remarkable performance surely can be attained if attention mechanism or phase estimation (Xu et al., 2020) is utilized in this method.

**Table 4:** Averaged rank-1 accuracies (%) on CASIA-B using setting LT, excluding identical-view cases.

Gallery NM#1-4	Probe views											
Probe CL#1-2	0°	18°	36°	54°	72°	90°	108°	126°	144°	162°	180°	Mean
Wu et al. (2017)	37.7	57.2	66.6	61.1	55.2	54.6	55.2	59.1	58.9	48.8	39.4	54.0
Zhang et al. (2019)	42.1	–	–	70.7	–	70.6	–	69.4	–	–	–	63.2
Chao et al. (2019)	61.4	75.4	80.7	77.3	72.1	70.1	71.5	73.5	73.5	68.4	50.0	70.4
Huang et al. (2020)	64.7	79.4	84.1	80.4	73.7	72.3	75.0	78.5	77.9	71.2	57.0	74.0
Hou et al. (2020)	70.6	82.4	85.2	82.7	79.2	76.4	76.2	78.9	77.9	78.7	64.3	77.5
Li et al. (2020b)	**78.2**	81.0	82.1	82.8	**80.3**	**76.9**	75.5	77.4	72.3	73.5	**74.2**	77.6
Li et al. (2020b)	70.7	**85.5**	**86.9**	83.3	77.1	72.5	76.9	82.2	**83.8**	**80.2**	66.5	**78.7**
Sepas-Moghaddam et al. (2021)	63.4	77.3	80.1	79.4	72.4	69.8	71.2	73.8	75.5	71.7	62.0	72.4
Sepas-Moghaddam & Etemad (2021)	65.8	80.7	82.5	81.1	72.7	71.5	74.3	74.6	78.7	75.8	64.4	74.7
Yao et al. (2021c)	64.2	80.9	83.0	79.5	74.3	69.1	74.8	78.5	81.0	77.0	60.3	74.8
**Ours**	68.3	83.3	86.3	**83.8**	77.8	76.1	**81.8**	**86.0**	83.1	78.0	59.3	78.5

**Notes.**

The first and second highest scores are represented by bold and underline, respectively.

To sum up, these comparison experiments on CASIA Gait Dataset B have certified that our proposed hybrid feature learning method is more feasible and effective when approaching this cloth-changing gait recognition problem. Compared with other gait recognition methods, this proposed method has performed a more remarkable result on this dataset for recognizing gaits across varying dressing styles.

### Comparison experiments on OU-ISIR treadmill gait dataset B

**Figure 5:**
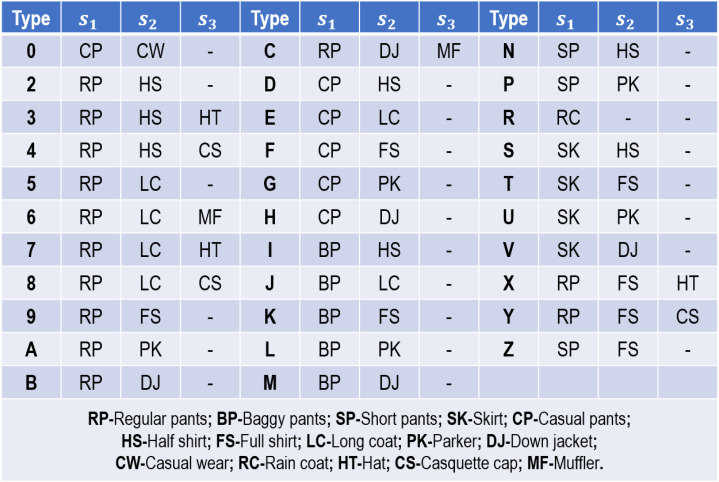
Thirty-two clothing combinations used in OU-ISIR Treadmill Dataset B.

As far as we know, OU-ISIR Treadmill Gait Dataset B (Makihara et al., 2012) has the maximum clothing conditions (Deng & Wang, 2018). It collects gait sequences from 68 persons in 32 clothing combinations, and each person in each clothing combination is recorded twice on the same day. Figure 5 presents the 32 clothing combinations used in this dataset. Given the varying clothing combinations, it is appropriate for us to validate the robustness and effectiveness of our proposed method on this dataset.

In this comparison experiment, our training set is assembled by the first sequence of each person in 32 clothing combinations, thereby 2,176 sequences contained. In our evaluation stage, 32 testing sets are respectively formed by the remaining sequences according to their clothing combinations. Table 5 reveals the comparison results for our proposed method with three other cloth-changing gait recognition methods. It can seen from this table that our proposed method enables to attain the accuracy of 100% in all clothing combinations, which verifies its strong robustness and effectiveness against varying clothing variations.

**Table 5:** Comparison on OU-ISIR Treadmill Dataset B by accuracies (%).

Probe set type	**Ours**	Yao et al. (2021c)	Anusha & Jaidhar (2019)	Deng & Wang (2018)
0	**100.0**	99.7	94.0	**100.0**
2	**100.0**	**100.0**	93.5	**100.0**
3	**100.0**	**100.0**	91.6	**100.0**
4	**100.0**	**100.0**	94.1	98.5
5	**100.0**	**100.0**	94.5	94.1
6	**100.0**	**100.0**	92.0	91.2
7	**100.0**	**100.0**	94.2	94.1
8	**100.0**	**100.0**	94.5	94.1
9	**100.0**	**100.0**	92.0	97.1
A	**100.0**	**100.0**	91.6	91.2
B	**100.0**	99.9	88.2	95.6
C	**100.0**	**100.0**	94.5	94.1
D	**100.0**	**100.0**	92.0	**100.0**
E	**100.0**	**100.0**	91.5	91.2
F	**100.0**	**100.0**	93.1	**100.0**
G	**100.0**	99.8	89.1	98.5
H	**100.0**	**100.0**	95.0	94.1
I	**100.0**	**100.0**	98.5	98.5
J	**100.0**	**100.0**	91.5	91.2
K	**100.0**	**100.0**	87.5	98.5
L	**100.0**	**100.0**	90.0	**100.0**
M	**100.0**	**100.0**	97.5	97.1
N	**100.0**	**100.0**	85.5	**100.0**
P	**100.0**	**100.0**	91.1	**100.0**
R	**100.0**	**100.0**	86.2	88.2
S	**100.0**	**100.0**	89.1	95.6
T	**100.0**	**100.0**	95.0	94.1
U	**100.0**	**100.0**	95.5	94.1
V	**100.0**	**100.0**	91.6	91.2
X	**100.0**	**100.0**	90.1	**100.0**
Y	**100.0**	**100.0**	89.0	**100.0**
Z	**100.0**	**100.0**	87.2	98.5

**Notes.**

The first and second highest scores are represented by bold and underline, respectively.

This experiment shows that no matter how significantly people alter their dressing styles, our proposed method can always effectively approach their changing styles and achieve a remarkable gait recognition performance. Made up of three sub-networks aiming to extract spatial and temporal features independently, our proposed method can offer a thorough gait description for each walking person. Also, given that for gait recognition local subtle features always prove more discriminative than global semantic features, in our method a sub-network is specifically proposed to grasp features from local subtle differences. Above all, compared with other gait recognition methods, our proposed hybrid feature learning method illustrates more capabilities for addressing this cloth-changing gait recognition problem. As long as a dressing style is involved in the training phase, it surely can be recognized in the following evaluation phase.

### Ablation experiments on CASIA gait dataset B

#### Effectiveness of different input frames

Table 6 shows the accuracy of different input frame numbers, and in this table (*a*, *b*) denotes the numbers of frames used for extracting spatial and temporal features respectively.

**Table 6:** Effectiveness of different input frames.

Input frame number	Accuracy (%)
(5, 5)	23.1
(10, 10)	73.8
(15, 15)	77.0
(20, 20)	77.3
(25, 25)	78.2
(30, 30)	78.5
(35, 35)	77.9

As presented in Table 6, the accuracy first monotonically rises as input frame numbers increase. This accuracy improvement first starts sharply and later levels off. Generally, the critical value is 25, and it is in accord with the frame number that a complete gait cycle normally has.

#### Effectiveness of different sub-networks

Table 7 compares the performance of the three sub-networks utilized in our method. It can be seen that the proposed hybrid spatio-temporal features have obtained the best result through concatenating these three sub-networks together. Besides, we can also find that features of the second sub-network, *i.e.*, *f*_*sg*_, cause a more significant influence on our hybrid gait features.

**Table 7:** Effectiveness of different sub-networks.

Feature component	Accuracy (%)
*f*_*sl*_	68.4
*f*_*sg*_	77.9
*f*_*t*_	69.6
*f*_*sl*_⨁*f*_*sg*_⨁*f*_*t*_	**78.5**

## Conclusion

Clothing variations have a significant influence on image/video-based gait recognition, and the performance can be sharply decreased if the probe and gallery gaits are no longer in a similar condition. Hence, a robust hybrid part-based spatio-temporal feature learning method was proposed in this article for gait recognition to approach the cloth-changing problem. First, each human body was divided into two parts, the affected parts and the non/less unaffected parts. After that, a well-designed network was proposed in this paper to formulate our required hybrid features from the divided non/less unaffected body parts. This network consists of three sub-networks, aiming to create robust features independently. Each sub-network emphasizes the individual aspects of gait, thus a potential hybrid gait feature was formulated through their concatenation. For example, because for gait recognition local detailed features prove more discriminative than global semantic features, one sub-network was specifically designed in our method to extract spatial features from local subtle areas. Moreover, given that temporal information can be deemed as complement to enhance the gait recognition performance, in our network one sub-network was also specifically proposed to extract the temporal relationship among successive short-range frames. The efficiency and effectiveness of our proposed method have been verified on CASIA Gait Dataset B and OU-ISIR Treadmill Gait Dataset B. The relevant experiments illustrate that this proposed hybrid feature learning method can always achieve a prominent result for gait recognition when handling the challenging cloth-changing problem.

## Supplemental Information

10.7717/peerj-cs.996/supp-1 Supplemental Information 1 Source code

## Additional Information and Declarations

### Data Availability

The following information was supplied regarding data availability: The code is available in the Supplementary File. The CASIA B dataset is available at: http://www.cbsr.ia.ac.cn/english/Gait%20Databases.asp. The OU-ISIR B dataset is available at: http://www.am.sanken.osaka-u.ac.jp/BiometricDB/GaitTM.html.
